# Supplementary material for: Splice-Junction-Based Mapping of Alternative Isoforms in the Human Proteome
Source: Cell Rep. Author manuscript; Available in PMC 2020 Jan 15. (PMC6961840; doi:10.1016/j.celrep.2019.11.026)
Supplement: 2 [file NIHMS1546469-supplement-2.zip › DF1/PXD010154/Thyroid/Thyroid_49_NARS_MIQVSQSQNVQK.pdf]

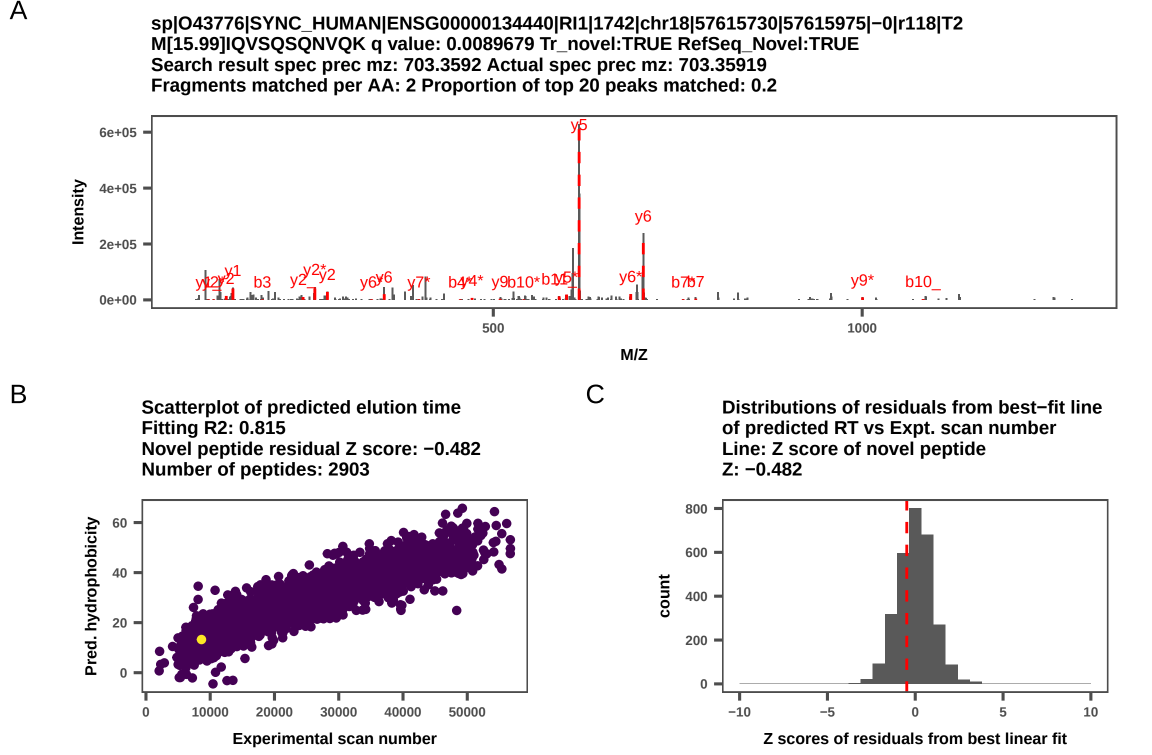
A
sp|O43776|SYNC_HUMAN|ENSG00000134440|RI1|1742|chr18|57615730|57615975|−0|r118|T2
M[15.99]IQVSQSQNVQK q value: 0.0089679 Tr_novel:TRUE RefSeq_Novel:TRUE
Search result spec prec mz: 703.3592 Actual spec prec mz: 703.35919
Fragments matched per AA: 2 Proportion of top 20 peaks matched: 0.2
Intensity
6e+05
4e+05
2e+05
0e+00
y5
y6
y1
b3
y2*
y2
y7*
y9*
b10_
M/Z
500
1000
B
Scatterplot of predicted elution time
Fitting R2: 0.815
Novel peptide residual Z score: −0.482
Number of peptides: 2903
Pred. hydrophobicity
60
40
20
0
0
10000
20000
30000
40000
50000
Experimental scan number
C
Distributions of residuals from best−fit line
of predicted RT vs Expt. scan number
Line: Z score of novel peptide
Z: −0.482
count
800
600
400
200
0
−10
−5
0
5
10
Z scores of residuals from best linear fit
